# Effects of Antiretroviral Therapy to Prevent HIV Transmission to Women in Couples Attempting Conception When the Man Has HIV Infection — United States, 2017

**DOI:** 10.15585/mmwr.mm6632e1

**Published:** 2017-08-18

**Authors:** John T. Brooks, Jennifer F. Kawwass, Dawn K. Smith, Dmitry M. Kissin, Margaret Lampe, Lisa B. Haddad, Sheree L. Boulet, Denise J. Jamieson

**Affiliations:** ^1^Division of HIV/AIDS Prevention, National Center for HIV/AIDS, Viral Hepatitis, STD, and TB Prevention, CDC; ^2^Division of Reproductive Health, National Center for Chronic Disease Prevention and Health Promotion, CDC; ^3^Division of Reproductive Endocrinology and Infertility, Department of Gynecology and Obstetrics, Emory University School of Medicine, Atlanta, Georgia.

Existing U.S. guidelines recommend that men with human immunodeficiency virus (HIV) infection should achieve virologic suppression* with effective antiretroviral therapy (ART) before attempting conception (*1*). Clinical studies have demonstrated that effective ART profoundly reduces the risk for HIV transmission (*2*–*4*). This information might be useful for counseling couples planning a pregnancy in which the man has HIV infection and the woman does not (i.e., a mixed HIV-status couple, often referred to as a serodiscordant couple). 

The risk for male-to-female sexual transmission of HIV in the absence of any prevention measures is estimated to be approximately 8 per 10,000 episodes of condomless intercourse (95% confidence intervals = 6–11) (*5*). Three multinational studies, HPTN 052 (*2*), PARTNER (*3*), and Opposites Attract (*4*), have provided data regarding the effectiveness of suppressing HIV replication with ART to reduce the risk for sexual HIV transmission. These studies followed approximately 3,000 sexually active mixed HIV-status couples over many years while they did not use condoms. The PARTNER and Opposites Attract studies quantified the extent of sexual exposure; 548 heterosexual couples (269 [49%] with a male HIV-infected partner) and 658 male-male couples from 14 European countries, Australia, Brazil, and Thailand engaged in >74,000 condomless episodes of vaginal or anal intercourse during >1,500 couple-years of observation (*3*,*4*). All three studies observed no HIV transmission to the uninfected partner while the partner with HIV was virologically suppressed with ART (*2*–*4*).

Recent studies have shown that men taking ART who have no detectable HIV RNA in their peripheral blood can occasionally have HIV genetic material detected in their semen (*6–8*). As many as 25% of men have had HIV RNA detected in semen after 3 months of viral suppression (*6*). After 4 or more months of suppression, reported detection rates in semen have been 5%–6% (*8*). In these studies, semen HIV RNA concentrations were 59–2,560 copies/mL (*6–8*). It is not known whether such detection represents the presence of replicating virus at sufficient concentration to transmit infection. HPTN 052, PARTNER, and Opposite Attract have not reported data on HIV RNA detection in semen; however, in the context of the above-cited information, it is possible HIV RNA could have been present in some semen specimens but that concentrations of replication competent virus were insufficient to transmit infection (*2–4*).

Mixed HIV-status couples attempting conception can also reduce the risk for sexually transmitting HIV by decreasing the frequency of sexual contact and limiting condomless intercourse to the time of ovulation. Preexposure prophylaxis (PrEP), a highly effective HIV prevention method in which the partner without HIV takes antiretrovirals in advance of potential HIV exposure (*9*), can also reduce the risk for a woman who is attempting conception with an HIV-infected man, especially if his viral load is not known or is detectable (*1*). Semen processing with subsequent intrauterine insemination (IUI) or in vitro fertilization (IVF) also significantly and substantially reduces transmission of HIV from men to women (*10*). For some couples, semen processing combined with IUI or IVF might be an option, especially if fertility treatment is needed or if the man’s HIV viral load cannot be fully suppressed. The extent to which any of these preventive interventions further decreases HIV risk below that associated with viral suppression and an undetectable viral load is unknown.

It is important that health care providers regularly assess mixed HIV-status couples’ plans for conception. Considering factors such as risk tolerance, personal health, costs, and access to health care services, providers can help couples make the best decision for their personal circumstances.

## Footnotes

* Usually defined as a confirmed HIV RNA level <200 copies/mL or below the lower limits of detection of an ultrasensitive assay.
